# Prescription characteristics associated with fall-related injury risk among older adults prescribed benzodiazepines: a cohort study

**DOI:** 10.1186/s12877-022-03497-3

**Published:** 2022-10-26

**Authors:** Donovan T. Maust, Amy S. B. Bohnert, Julie Strominger, Neil Alexander, Lillian Min, Geoffrey J. Hoffman, Jason E. Goldstick

**Affiliations:** 1 Injury Prevention Center, University of Michigan, Ann Arbor, MI United States; 2 Department of Psychiatry, University of Michigan Medical School, Ann Arbor, MI United States; 3 Center for Clinical Management Research, VA Ann Arbor Healthcare System, 2800 Plymouth Rd, NCRC 016-226W, Ann Arbor, MI 48109 United States; 4 Department of Anesthesiology, University of Michigan Medical School, Ann Arbor, MI United States; 5 Department of Internal Medicine, University of Michigan Medical School, Ann Arbor, MI United States; 6 Geriatric Research Education Clinical Center, Virginia Ann Arbor Healthcare System, Ann Arbor, MI United States; 7 University of Michigan School of Nursing, Ann Arbor, MI United States; 8 Department of Emergency Medicine, University of Michigan Medical School, Ann Arbor, MI United States

**Keywords:** Medication, Hip fracture, Epidemiology

## Abstract

**Background:**

Benzodiazepines (BZD) are widely prescribed to older adults despite their association with increased fall injury. Our aim is to better characterize risk-elevating factors among those prescribed BZD.

**Methods:**

A retrospective cohort study using a 20% sample of﻿ Medicare beneficiaries with Part D prescription drug coverage. Patients with a BZD prescription (“index”) between 1 April 2016 and 31 December 2017 contributed to incident (*n*=379,273) and continuing (*n*=509,634) cohorts based on prescriptions during a 6-month pre-index baseline. Exposures were index BZD average daily dose and days prescribed; baseline BZD medication possession ratio (MPR) (for the continuing cohort); and co-prescribed central nervous system-active medications. Outcome was a treated fall-related injury within 30 days post-index BZD, examined using Cox proportional hazards adjusting for demographic and clinical covariates and the dose prescribed.

**Results:**

Among incident and continuing cohorts, 0.9% and 0.7% experienced fall injury within 30 days of index. In both cohorts, injury risk was elevated immediately post-index among those prescribed the lowest quantity: e.g., for <14-day fill (ref: 14-30 days) in the incident cohort, risk was 37% higher the 10 days post-fill (adjusted hazard ratio [HR] 1.37 [95% confidence interval [CI] 1.19-1.59]). Risk was elevated immediately post-index for continuing users with low baseline BZD exposure (e.g., for MPR <0.5 [ref: MPR 0.5-1], HR during days 1-10 was 1.23 [CI 1.08-1.39]). Concurrent antipsychotics and opioids were associated with elevated injury risk in both cohorts (e.g., incident HRs 1.21 [CI 1.03-1.40] and 1.22 [CI 1.07-1.40], respectively; continuing HRs 1.23 [1.10-1.37] and 1.21 [1.11-1.33]).

**Conclusions:**

Low baseline BZD exposure and a small index prescription were associated with higher fall injury risk immediately after a BZD fill. Concurrent exposure to antipsychotics and opioids were associated with elevated short-term risk for both incident and continuing cohorts.

**Supplementary Information:**

The online version contains supplementary material available at 10.1186/s12877-022-03497-3.

## Background

Falls are the leading cause of unintentional injury among adults ≥65 years old in the U.S., responsible for 2.2 million emergency department visits in 2016 [1], while fall-related mortality among older adults more than doubled from 2000 to 2016 [2]. Benzodiazepines (BZDs) are among the medication classes most strongly associated with falls and fall-related injury [3]. Despite professional guidelines that recommend avoiding prescribing BZDs to older adults due to associated safety risks [4, 5], prescribing has not decreased [6], with annual use reported by nearly 13% of older adults in the U.S [7].

While the safety risks associated with BZD use relative to non-use are known, data characterizing risks among those prescribed BZD are limited. There are some older adults for whom BZD use is warranted, such as for patients with treatment-resistant anxiety [8, 9] or refractory epilepsy [4]. Expert opinion and professional guidelines suggest that short-term or intermittent BZD use is safer [8, 10, 11], yet few data support this. Brief or intermittent BZD exposure may mean patients more slowly develop physiological tolerance [12], yielding sedative effects—and therefore fall risk—more pronounced than with regular consumption. While there is an increased risk of fall injury associated with higher burden of psychotropic and opioid medications [13–15], risks of specific combinations with BZD are unclear. In the face of the continued relatively high level of BZD prescribing to adults, characterizing who, among those prescribed BZD, is at increased risk of injury may help inform clinicians’ prescribing and deprescribing decisions.

In this study, we use a national random sample of Medicare beneficiaries (Medicare is national, government-sponsored insurance in the U.S. for those ≥65 as well as permanently disabled individuals) to identify characteristics associated with risk of a treated fall-related injury within 30 days following a BZD prescription fill, focusing on characteristics of the BZD exposure (e.g., average daily dose, days’ supply) as well as other prescription central nervous system-active (CNS-active) medications. We hypothesized that fewer days of BZD exposure would be associated with higher risk, while along with opioids, co-prescriptions of antipsychotics and antiepileptics would be associated with increased risk of injury.

## Methods

### Study population

For this retrospective cohort study, we began with a 20% random sample of Medicare beneficiaries with ≥6 months of continuous insurance coverage including prescription drugs between October 2015 and December 2017 (see Figure S1 for further details). Because this analysis focused on risk among those prescribed a BZD, we identified all BZD fills (“treatment episode”) during periods of continuous coverage that occurred between 1 April 2016 and 31 December 2017. We then limited treatment episodes to those BZD fills preceded by at least 6 months of continuous insurance coverage—which served as the baseline for deriving clinical characteristics—and where the beneficiary was 65+ on the day of the fill.

To assign treatment episodes to the incident or continuing cohort, we examined BZD fills during each episode’s 6-month baseline. Where the baseline contained no BZD fills, the episode was assigned to the incident cohort; if a beneficiary had multiple potential incident treatment episodes, only the first was used. Where the baseline contained ≥1 BZD fill(s), the episode was assigned to the continuing cohort; if a beneficiary had multiple potential continuing treatment episodes, only the first was used. The date of the treatment-episode-defining BZD fill was considered the index date. Finally, because a prior fall is one of the strongest risk factors for a future fall—and therefore most older adults that experience fall injury should not be prescribed a BZD [16]—we excluded those who had a fall-related injury during their respective baselines. Our goal was to inform more clinically ambiguous decision-making among those without evidence of a prior fall.

Given the 6-month baseline requirement, the first possible index date for a beneficiary was April 1, 2016, with a baseline period beginning October 1, 2015 (i.e., the start of *ICD-10-CM* use in the U.S.).

### Outcome

Each cohort was followed from the day of the index BZD prescription until the earliest of the following: fall-related injury event, death, change in insurance coverage, 30 days after the index prescription, or end of study data (December 31, 2017). We identified fall-related injury events (“fall injury” hereafter) using the claims-based balanced algorithm developed and validated by Min et al. [17], which identifies fall injuries resulting in acute or severe nonemergency encounters.

### Exposures

For both cohorts, our primary exposures of interest were characteristics of the prescribed BZD as well as prescriptions of other common CNS-active medications (Table S1). For the index BZD, we derived average daily dosage (<1, 1-1.99, or 2+ in lorazepam-equivalent [lor-eq] mg/day hereafter [18]) and days’ supply (<14, 14-30, or 30+ days). For the continuing BZD cohort, we also computed the baseline BZD medication possession ratio (MPR), calculated as the sum of BZD days prescribed during baseline divided by 180 days (i.e., the 6-month baseline; Figure S2), categorized as <0.5, 0.5-1, or >1. These variables were categorized based on clinically meaningful cut-points similar to prior analyses [19, 20].

For the other exposures of interest, we used prescription fills and days prescribed during the baseline to classify each cohort member, on the index date, as a current (i.e., exposure that covered the index date), former (i.e., exposure during baseline that did not include the index date), or never use (no days of exposure during baseline or the index date) of: antidepressants, antiepileptics, antipsychotics, opioids, and non-benzodiazepine benzodiazepine receptor agonists (i.e., ‘z-drugs’).

### Other characteristics

We controlled for age, sex, race/ethnicity, low-income subsidy, rurality, and season. Race/ethnicity was derived using the Research Triangle Institute race code variable from the Medicare Master Beneficiary Summary File. Low-income subsidy was set to present if, during baseline, a beneficiary had ≥1 month where they were eligible for or enrolled in the Part D low-income subsidy. Patient zip code was used to identify census division and derive rurality (along with Rural-Urban Continuum Codes); we also included season and an interaction between census division and season.

We used baseline health care encounters to account for clinical characteristics potentially associated with both the exposures of interest and fall injury [20, 21], including dementia, osteoarthritis, stroke, substance use disorders, urinary incontinence, and medications to treat hypertension and Parkinson’s disease. In addition to these specific factors potentially associated with fall injury, we used the Elixhauser comorbidity index [22]—a count of 30 conditions—to capture overall burden of medical comorbidity. Finally, frailty is an age-related condition of decline in physiological function and increased susceptibility to stressors [23] that is associated with adverse outcomes including mortality and falls. We used a claims-based frailty index, which is a weighted scale including medical conditions and durable medical equipment (e.g., walking aids), to capture this additional confounder [24]. The scale of the frailty index is 0 to 1, which we dichotomized as not frail (<0.2) and frail (≥0.2) [25].

### Statistical analysis

We first summarized cohort characteristics as well as the index BZD prescribed. To visually explore the probability of fall-risk injury by select characteristics, we generated Kaplan-Meier survival curves within each cohort, stratified by BZD exposure variables of interest (MPR, days’ supply, average daily dose). We used log-rank tests to test if survival functions were equal across levels of the given exposure variable.

We then fit Cox proportional hazards models for each cohort to examine factors associated with fall injury following BZD use, adjusting for the other covariates outlined above. We determined if the assumption of proportional hazards was met by examining plots of Schoenfeld residuals versus time. To account for those lost to follow up due to death or loss of coverage (“artificial censoring”), we used inverse probability weighting to recover estimates consistent with a population without artificial censoring [26]. Specifically, we: 1) fit a logistic regression model where the outcome was not being artificially censored; 2) used the predicted values from that model to construct weights (1 / [predicted probability of not being censored due to death or loss of coverage]); and 3) applied those weights to a Cox regression model using only those who were not artificially censored. Across both cohorts, weights ranged from 1.001 to 96.03; the 99^th^ percentile was 1.91. Robust standard errors were used to account for the use of dropout weights.

Because of observed imbalance in covariates across categories of days’ supply (i.e., <14, 14-30, and >30 days’ supply), we conducted a sensitivity analysis for both incident and continuing cohorts using generalized propensity scores to account for these observed imbalances (see Methods S1 for more information).

Alpha was set at 0.05 and all tests were two-sided, with analyses conducted using SAS 9.4 and R version 4.1.0. The analysis was approved by the Michigan Medicine IRB and a STROBE checklist for cohort studies is provided in the online supplement.

## Results

### Incident cohort

The incident cohort included 379,273 older adults newly prescribed a BZD; 68.9% were female, 87.2% were non-Hispanic white (Table 1). The most common non-BZD concurrent medication exposures of interest were antidepressants (33.9%), opioids (19.9%), and antiepileptics (14.7%). The mean average daily BZD dose of the index prescription was 1.5 (standard deviation [SD] 1.5) lor-eq mg/day, while days’ supply was 23.0 (SD 21.4). Alprazolam was most commonly filled (Table 2; 35.2%), followed by lorazepam (32.5%) and diazepam (16.3%).

**Fig. 1:**
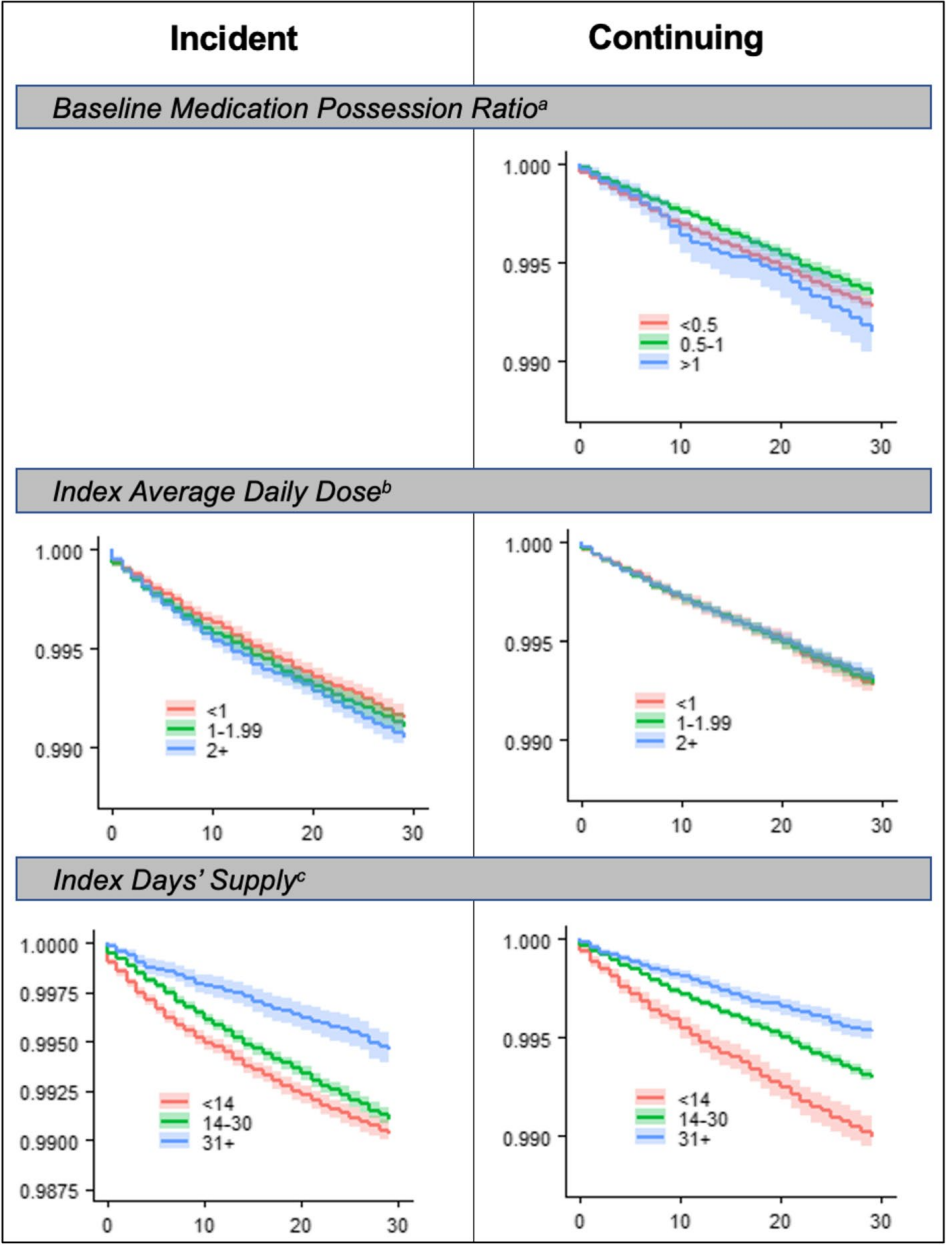
Kaplan Meier Survival Curves of Fall-Related Injury Events Among Incident and Continuing BZD Users by BZD Exposures. MPR, medication possession ratio; BZD, benzodiazepine. For each panel, the y-axis presents the survival probability and x-axis is days from index BZD prescription. ^**a**^ MPR log-rank (LR) test *p*=0.003. ^**b**^ Average daily dose LR test: *p*=0.06 (incident); *p*=0.34 (continuing). ^**c**^ Days’ supply LR test: *p*<.001 (incident); *p*<.001 (continuing)

Within 30 days of the index BZD, 0.9% of the incident cohort experienced a fall injury. Figure 1 presents the Kaplan Meier survival distributions based on index BZD exposure. There was not a relationship between average daily dose and probability of survival (log-rank [LR] test *p*=0.06), while there was based on index days’ supply, with distributions suggesting a lower event-free survival probability among those prescribed fewer days (LR test *p*<.001).

**Fig. 2:**
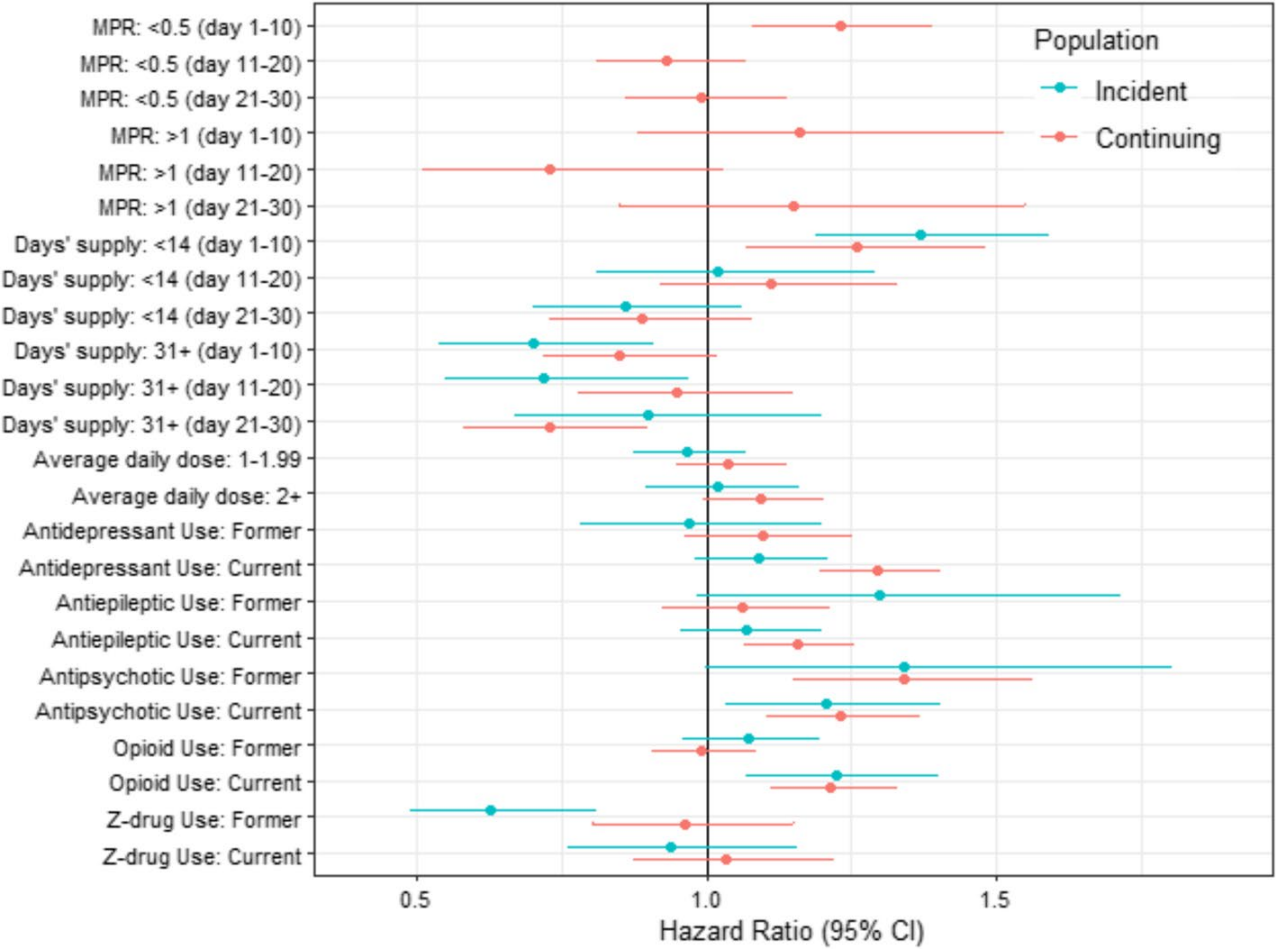
Cox Proportional Hazards Model of Fall-Related Injury Events Following Incident and Continuing Benzodiazepine Use: Benzodiazepine and Other Prescription Medication Characteristics. MPR, medication possession ratio; CI, confidence interval; BZD, benzodiazepine. Adjusted for sociodemographic, clinical characteristics, census division, seasonality, and the interaction between census division and seasonality (full model presented in Table S2). The reference groups for BZD exposure are as follows: MPR (0.5-1); Average daily dose (<1 lorazepam-equivalent mg/day); and Days’ supply (14-30 days). The reference group for specific medication classes (e.g., Antidepressant use) is never

Figure 2 presents associations between medication exposures of interest and adjusted hazards of fall injury within 30 days (unadjusted and fully adjusted results in Table S2). In the adjusted models, the dosage of the index BZD was not associated with risk, but days’ supply was. Because plots of Schoenfeld residuals versus time indicated non-proportional hazards for days’ supply, days’ supply was included as a time-dependent coefficient using step functions, which allows for a different coefficient (i.e., adjusted hazard ratio [aHR]) for different time intervals. For ease of interpretation and based on Schoenfeld plots, we used time intervals of 10 days (e.g., days 1-10, 11-20, and 21-30). Therefore, relative to the reference group (i.e., those who received an index BZD of 14-30 days), we computed three aHRs each (corresponding to risk during days 1-10, 11-20, and 21-30) for those who received <14 and 31+ days.

During the first 10 days of follow-up, those in the smallest index prescription group (i.e., <14 days) had 37% higher risk of fall injury (aHR 1.37 [CI 1.19-1.59]) than those in the reference group (14-30 days). Those who received 31+ days had lower risk of injury than the reference group during days 1-10 (aHR 0.70 [CI 0.54-0.91]) and 11-20 (aHR 0.72 [CI 0.55-0.97]), but risk was no longer significantly lower after day 20.

Among co-prescribed medications for those filling an incident BZD, concurrent exposure to antipsychotics and opioids were associated with increased fall injury risk (aHR 1.21 [CI 1.03-1.40]; 1.22 [CI 1.07-1.40], respectively), while former Z-drug exposure was associated with lower risk of injury relative to those without (aHR 0.63 [CI 0.49-0.81]).

### Continuing cohort

The continuing cohort included 509,634 older adults prescribed an index BZD; 70.7% were female and 87.8% were non-Hispanic white (Table 1). The most common non-BZD concurrent medication exposures of interest were antidepressants (44.0%), opioids (22.7%), and antiepileptics (18.3%). The mean medication possession ratio (MPR) was 0.5 (standard deviation [SD] 0.4); the index prescription average daily BZD dose was 1.9 (SD 1.77) lor-eq mg/day and days’ supply was 33.9 (SD 22.9). Alprazolam was most commonly filled (Table 2; 36.6%), followed by lorazepam (28.8%) and clonazepam (16.9%).

Within 30 days of the index BZD, 0.7% of the continuing cohort experienced a fall injury. Figure 1 presents the Kaplan Meier survival distributions based on index BZD exposure. Injury-free survival probability varied significantly by baseline MPR, highest among those in the intermediate MPR group (LR test *p*=0.003). There was no relationship between average daily dose and survival probability (LR test *p*=0.34), though there was based on index days’ supply, with plots suggesting a lower injury-free survival probability among those prescribed fewer days (LR test *p*<.001).

Figure 2 presents associations between medication exposures of interest and adjusted hazards of fall injury within 30 days (unadjusted and fully adjusted results in Table S2). Similar to the incident cohort, plots of Schoenfeld residuals versus time suggested non-proportional hazards for days’ supply and for MPR. We followed the same steps as for the incident cohort and allowed aHRs for days’ supply and MPR to vary over time. Compared to those with baseline MPR of 0.5-1 (reference group), during the first 10 days after the index BZD fill, those with MPR <0.5 (i.e., they filled BZD prescriptions that covered <50% of baseline days) had 23% higher injury risk than the reference group (aHR 1.23 [CI 1.08-1.39]); after day 10, their risk was similar to reference. During the first 10 days of follow up, those in the smallest index prescription group (i.e., <14 days) had 26% higher risk of fall injury (aHR 1.26 [CI 1.07-1.48]) than the reference group (14-30-day fill); risk was then similar after day 10. Those who received 31+ days had a lower risk of injury than reference, though not until days 21-30 (aHR 0.73 [0.58-0.90]).

Among co-prescribed medications for those prescribed an incident BZD, concurrent exposure to other medications except Z-drugs were all associated with increased risk of injury: antidepressants (aHR 1.30 [CI 1.20-1.40]); antiepileptics (aHR 1.16 [CI 1.06-1.26]); antipsychotics (aHR 1.23 [CI 1.10-1.37]); and opioids (aHR 1.21 [CI 1.11-1.33]).

In sensitivity analysis where we accounted for imbalance in characteristics across days’ supply (Table S3), we found that results related to the smallest days’-supply category (i.e., <14 days) did not substantively change. However, those with prescriptions of 31+ days no longer had periods of decreased risk of fall-related injury (see Supplementary Methods and Results for full details).

## Discussion

In this large national study of Medicare beneficiaries with Part D prescription drug coverage, 0.9% of those with a new BZD prescription and 0.7% of those with ongoing BZD treatment experienced a fall injury within 30 days of a BZD prescription fill. Features of the prescribed BZD were associated with risk: those prescribed the smallest supply of pills (i.e., <14 days) were at increased risk within the first 10 days following the index fill, as were those with the lowest level of baseline BZD exposure among the continuing users. Finally, concurrent antipsychotic and opioid prescriptions were associated with increased risk of injury in both incident and continuing cohorts.

Increased risk of fall injury associated with BZD use has been recognized for over 30 years [27, 28], yet prescribing to older adults remains high [6], and BZD prescriptions are rarely changed even after a fall [29]. Prior analyses of the association between BZD regimen and risk of injury have focused on the risk of use versus non-use [19, 20, 30], but in the face of such persistent use, clinicians and patients may benefit from a more nuanced characterization of risk among those prescribed BZD. In considering BZD prescriptions compared to non-use, prior analyses have demonstrated increased risk of hip fracture associated with higher dosages, including heightened risk during the first two weeks of the index prescription [19, 20, 31].

Our findings extend this work by demonstrating that, as hypothesized among cohorts entirely consisting of BZD users, early fall injury risk is most pronounced among those prescribed small quantities and, among those with continuing use, those with the lowest levels of baseline BZD exposure (i.e., the lowest medication possession ratio). While perhaps counterintuitive, such limited exposure may be less safe due to heightened sensitivity to the sedating effects of the medication before physiological tolerance has developed [12]. These findings may also be related to residual confounding, with clinicians prescribing smaller quantities to patients that they perceive to be at high risk. However, when accounting for observed differences between groups based on the days’ supply (e.g., among the continuing cohort, the <14 days’-supply group was more frail), the findings remained. Regardless of whether the findings reflect causality or confounding, they run counter to current recommendations for use [8, 10, 11]: Clinicians should be aware that patients to whom they prescribe limited BZD quantities with intermittent consumption are at elevated risk of fall injury.

In both the incident and continuing cohorts, we demonstrated increased risk of fall injury among those with particular combinations of concurrently prescribed CNS-active medications. In the incident cohort, concurrent antipsychotic and opioid use had associated increased risk. The continuing cohort had elevated risk for antipsychotics and opioids, in addition to antidepressants and antiepileptics. Overall, these findings are consistent with a body of work demonstrating increased fall risk associated with higher burden of central nervous system active medications [14, 15, 32]. The one unexpected finding was a lower risk associated with prior exposure to Z-drugs among the incident BZD cohort. Given that these medications are benzodiazepine receptor agonists, it is possible that prior exposure would lead patients to develop some level of physiological tolerance, such that the effect of an incident index BZD would be less pronounced than among those who filled a BZD prescription without prior Z-drug exposure.

Our analysis has several limitations. This observational work allowed us to examine factors associated with elevated risk but does not establish causality. The associations of lower early injury risk with lower baseline use and a smaller index prescription may reflect residual confounding, with clinicians more cautiously prescribing to patients whom they correctly perceive to be at high risk. Our measures of medication exposure are based on prescription claims and may not reflect actual consumption, nor do they capture self-pay prescriptions. Finally, the analysis is limited to those in Medicare with prescription drug coverage and does not include those in Medicare Advantage (i.e., managed Medicare offered by commercial insurance companies who receive payments per enrolled Medicare beneficiary from the federal government), which limits the generalizability.

These limitations are balanced by the strengths, including analysis of a large, national cohort and including a measure of frailty, an important potential confounder not been previously considered in analyses of the association between BZD exposure and fall-related injury. Finally, to our knowledge, this is the first analysis of BZD-related harms that has considered how the pattern of consumption (e.g., medication possession ratio) may be associated with harms.

Clinically, what are providers to do? The American Geriatrics Society/Britis﻿h Geriatrics Society Clinical Practice Guideline for Prevention of Falls in Older Persons recommends annual fall risk screening for adults ≥65 [33], while a recent review of fall prevention for community-dwelling older adults highlighted medication review as a key component of fall risk assessment [34]. Somewhat paradoxically, however, in clinical practice CNS-active medications are generally not being reduced after older adults experience fall injury [29]. Based on our analysis, first it is important for clinicians to recognize—and counsel patients appropriately—that older adults who receive a short-term BZD prescription are not necessarily at lower risk of fall injury. Second, we found the majority of other CNS-active medications associated with increased risk of injury, with antipsychotics and opioids consistently associated for both incident and continuing users. This is consistent with work suggesting overall burden of CNS-active medication burden contributes to fall risk [15, 32], while the specific combinations may be less important. These prior analyses, both of CNS-active medications overall and BZD specifically, consistently demonstrate that higher dosages are associate with elevated fall risk. Therefore, if clinicians and their patients do not believe that the number of such medications can be reduced, they should endeavor to identify the lowest effective dosages possible. In addition, in patients that need combination CNS-active regimens, clinicians should aggressively consider other fall prevention interventions as reviewed by Phelan and Ritchey, including strength and balance training, home assessment and modification, and optimal management of other medications (e.g., antihypertensives, diuretics) [34], along with appropriate screening and treatment for osteoporosis [35].

## Conclusions

Clinicians continue to prescribe BZD widely to older adults, despite the known safety risks and growing toll of fall injury. While our analysis does not establish causality, we do identify characteristics of both the baseline pattern of BZD use, index BZD prescription, and concurrent medications that may be useful features for clinicians to consider as they try to prescribe safely. Perhaps most notably, we found that low levels of BZD exposure—both of baseline use over the prior 6 months for continuing users and of the index BZD prescription for both incident and continuing users—were associated with elevated fall injury risk during the 10 days immediately after the prescription fill. This analysis suggests important next steps for further investigation to isolate causal effects, focusing specifically on different patterns of BZD exposure and co-prescribing. In the interim, however, it is important for clinicians to consider that smaller prescriptions or limited use among their older patients is not associated with a lower risk of fall injury.

**Table 1:** Characteristics of Incident and Continuing Benzodiazepine Cohorts at Risk of Fall Events

**Characteristic, n (%)**	**Incident Cohort** ***N*=379,273**^**a**^	**Continuing Cohort** ***N*=509,634**^**b**^
*Sociodemographics*		
Sex		
Male	118,036 (31.1)	149,442 (29.3)
Female	261,237 (68.9)	360,192 (70.7)
Age		
65-74	202,666 (53.4)	283,473 (55.6)
75-84	119,955 (31.6)	153,270 (30.1)
85+	56,652 (14.9)	72,891 (14.3)
Race^c^		
Non-Hispanic White	330,901 (87.2)	447,637 (87.8)
Non-Hispanic Black	18,783 (5.0)	24,197 (4.7)
Hispanic	18,881 (5.0)	26,481 (5.2)
Asian/Pacific Islander	7,446 (2.0)	7,260 (1.4)
Other	3,262 (0.9)	4,059 (0.8)
Low-income subsidy^d^		
No	294,854 (77.7)	361,558 (70.9)
Yes	84,419 (22.3)	148,076 (29.1)
Rurality^e^		
Urban	331,191 (87.3)	437,221 (85.8)
Rural	48,082 (12.7)	72,413 (14.2)
*Clinical Characteristics*		
Elixhauser^f^		
0-1	104,851 (27.6)	131,720 (25.8)
2	66,511 (17.5)	94,097 (18.5)
3	57,384 (15.1)	81,665 (16.0)
4	44,194 (11.7)	62,659 (12.3)
5	32,454 (8.6)	45,360 (8.9)
6	22,933 (6.0)	31,578 (6.2)
7	16,251 (4.3)	21,471 (4.2)
8	11,618 (3.1)	14,598 (2.9)
9+	23,077 (6.1)	26,486 (5.2)
Dementia	46,734 (12.3)	60,218 (11.8)
Osteoarthritis	112,096 (29.6)	156,351 (30.7)
Stroke	22,986 (6.1)	27,777 (5.5)
Substance Use Disorder	28,353 (7.5)	49,498 (9.7)
Urinary incontinence	21,180 (5.6)	28,345 (5.6)
Antihypertensive	280,960 (74.1)	391,420 (76.8)
Parkinson’s disease medication	18,582 (4.9)	32,452 (6.4)
Frailty^g^		
Not frail	249,705 (65.8)	324,251 (63.6)
Frail	129,568 (34.2)	185,383 (36.4)
*BZD Characteristics*		
Medication possession ratio, %		
<0.5	n/a	320,176 (62.8)
0.5-1	n/a	172,766 (33.9)
>1	n/a	16,692 (3.3)
Average daily dose, lor-eq mg/day		
<1	110,981 (29.3)	109,554 (21.5)
1-1.99	165,125 (43.5)	196,560 (38.6)
2+	103,167 (27.2)	203,520 (39.9)
Days’ supply, days		
<14	144,816 (38.2)	59,415 (11.7)
14-30	201,315 (53.1)	371,159 (72.8)
31+	33,142 (8.7)	79,060 (15.5)
*Other Medication Use*		
Antidepressant		
Never	224,801 (59.3)	243,037 (47.7)
Former	25,828 (6.8)	42,305 (8.3)
Current	128,644 (33.9)	224,292 (44.0)
Antiepileptics		
Never	302,869 (79.9)	385,350 (75.6)
Former	20,517 (5.4)	30,931 (6.1)
Current	55,887 (14.7)	93,353 (18.3)
Antipsychotics		
Never	348,124 (91.8)	449,619 (88.2)
Former	8,679 (2.3)	16,107 (3.2)
Current	22,470 (5.9)	43,908 (8.6)
Opioids		
Never	237,245 (62.6)	286,972 (56.3)
Former	66,477 (17.5)	106,963 (21.0)
Current	75,551 (19.9)	115,699 (22.7)
Z-drugs		
Never	352,229 (92.9)	464,349 (91.1)
Former	11,001 (2.9)	19,454 (3.8)
Current	16,043 (4.2)	25,831 (5.1)

*BZD* Benzodiazepine, *SD* Standard deviation, *lor-eq* Lorazepam-equivalent

^a^0.9% had fall event; 93.3% censored at 30d after index, 3.4% for loss of coverage, 2.5% for death

^b^0.7% had fall event; 96.8% censored at 30d after index, 1.7% for loss of coverage, 0.7% for death

^c^Derived using the Research Triangle Institute race variable; race groups are mutually exclusive

^d^Considered present if a given beneficiary was enrolled or eligible in the Part D low-income subsidy for at least one month during the 6-month baseline period

^e^Derived using beneficiary state and county codes and Rural-Urban Continuum Codes

^f^Excludes substance use disorders and depression, which were captured separately

^g^Frailty dichotomized following Kim et al.[36]

**Table 2:** Index Benzodiazepine Prescribed to Incident and Continuing Benzodiazepine Users

	**N (%)**	
**Index BZD prescription**^**a**^	**Incident Cohort** ***N*=379,273**	**Continuing Cohort** ***N*=509,634**
Alprazolam	133,530 (35.2)	186,570 (36.6)
Lorazepam	123,142 (32.5)	146,991 (28.8)
Clonazepam	33,376 (8.8)	85,962 (16.9)
Diazepam	61,668 (16.3)	48,015 (9.4)
Temazepam	23,172 (6.1)	35,948 (7.1)
Clorazepate	1,992 (0.5)	4,480 (0.9)
Chlordiazepoxide	1,688 (0.4)	2,205 (0.4)
Triazolam	1,273 (0.3)	1,461 (0.3)
Oxazepam	340 (0.1)	1,003 (0.2)
Flurazepam	200 (0.1)	324 (0.1)

*BZD* Benzodiazepine

^a^Column percentages may sum to >100% due to some patients being prescribed >1 index BZD prescriptions on the same day. Only the top 10 BZDs are shown: estazolam, midazolam, and clobazam each accounted for ≤0.1% within each cohort

## Supplementary Information

**Additional file 1:** **Figure S1.** Flow Chart of the Study Population Examining Factors Associated with Fall Related Injury Events among Incident and Continuing Benzodiazepine Users. **Table S1.** Medications Contributing to Each Medication Class. **Figure S2.** Computing Medication Possession Ratio (MPR) for Continuing Benzodiazepine Users. **Table S2.** Characteristics Associated with Fall Related Injury Event Among Incident and Continuing Benzodiazepine Users. **Table S3.** Distribution of Characteristics of Incident and Continuing Benzodiazepine Users by Days’ Supply: Before Weighting.

## Data Availability

The data that support the findings of this study are available from the Centers for Medicare & Medicaid Services (CMS). Under the terms of a research investigator Data Use Agreement with CMS, individual investigators are strictly prohibited from making CMS data publicly available. Investigators can independently request the data used to conduct this analysis from CMS.
