# Supplementary material for: Prescription characteristics associated with fall-related injury risk among older adults prescribed benzodiazepines: a cohort study
Source: BMC Geriatr. 2022 Oct 26;22:824. doi: 10.1186/s12877-022-03497-3 (PMC9609287; doi:10.1186/s12877-022-03497-3)
Supplement: Supplementary file 1 — Additional file 1: Figure S1. Flow Chart of the Study Population Examining Factors Associated with Fall Related Injury Events among Incident and Continuing Benzodiazepine Users. Table S1. Medications Contributing to Each Medication Class. Figure S2. Computing Medication Possession Ratio (MPR) for Continuing Benzodiazepine Users. Table S2. Characteristics Associated with Fall Related Injury Event Among Incident and Continuing Benzodiazepine Users. Table S3. Distribution of Characteristics of Incident and Continuing Benzodiazepine Users by Days’ Supply: Before Weighting. [file 12877_2022_3497_MOESM1_ESM.docx]

### **Supplementary Material**

### **STROBE Statement.** Checklist for observational cohort studies

### **Figure S1.** Flow Chart of the Study Population Examining Factors Associated with Fall Related Injury Events among Incident and Continuing Benzodiazepine Users

## **Table S1.** Medications Contributing to Each Medication Class

**Figure S2.** Computing Medication Possession Ratio (MPR) for Continuing Benzodiazepine Users

## **Table S2.** Characteristics Associated with Fall Related Injury Event Among Incident and Continuing Benzodiazepine Users

## **Table S3.** Distribution of Characteristics of Incident and Continuing Benzodiazepine Users by Days’ Supply: Before Weighting

## **Supplementary Methods.** Examining the Association Between Days’ Supply and Fall-Related Injury Among Older Adults at Risk of Incident Fall-related Injury: Accounting for Imbalance in Characteristics Across Days’ Supply

## **Supplementary Results.**

## **Table S4.** Distribution of Characteristics of Incident Benzodiazepine Users by Days’ Supply: After Inverse Probability of Treatment Weighting

## **Table S5.** Distribution of Characteristics of Continuing Benzodiazepine Users by Days’ Supply: After Inverse Probability of Treatment Weighting

## **Table S6.** Association Between Days’ Supply and Fall-Related Injury After Accounting for Imbalance in Characteristics Across Days’ Supply and Dropout: Incident and Continuing BZD users

**STROBE Statement.** Checklist of items that should be included in reports of ***cohort studies***

|  | Item No | Recommendation | Page No |
| --- | --- | --- | --- |
| **Title and abstract** | 1 | (*a*) Indicate the study’s design with a commonly used term in the title or the abstract | 1 |
|  |  | (*b*) Provide in the abstract an informative and balanced summary of what was done and what was found | 2 |
| Introduction | | | |
| Background/rationale | 2 | Explain the scientific background and rationale for the investigation being reported | 2 |
| Objectives | 3 | State specific objectives, including any prespecified hypotheses | 3-4 |
| Methods | | | |
| Study design | 4 | Present key elements of study design early in the paper | 4 |
| Setting | 5 | Describe the setting, locations, and relevant dates, including periods of recruitment, exposure, follow-up, and data collection | 4 |
| Participants | 6 | (*a*) Give the eligibility criteria, and the sources and methods of selection of participants. Describe methods of follow-up | 4 |
|  |  | (*b*) For matched studies, give matching criteria and number of exposed and unexposed | n/a |
| Variables | 7 | Clearly define all outcomes, exposures, predictors, potential confounders, and effect modifiers. Give diagnostic criteria, if applicable | 4-7 |
| Data sources/ measurement | 8* | For each variable of interest, give sources of data and details of methods of assessment (measurement). Describe comparability of assessment methods if there is more than one group | 4-7 |
| Bias | 9 | Describe any efforts to address potential sources of bias | 7 |
| Study size | 10 | Explain how the study size was arrived at | Fig S1 |
| Quantitative variables | 11 | Explain how quantitative variables were handled in the analyses. If applicable, describe which groupings were chosen and why | 5 |
| Statistical methods | 12 | (*a*) Describe all statistical methods, including those used to control for confounding | 6,7 |
|  |  | (*b*) Describe any methods used to examine subgroups and interactions | n/a |
|  |  | (*c*) Explain how missing data were addressed | n/a |
|  |  | (*d*) If applicable, explain how loss to follow-up was addressed | n/a |
|  |  | (*e*) Describe any sensitivity analyses | 7 |
| Results | | |  |
| Participants | 13* | (a) Report numbers of individuals at each stage of study—eg numbers potentially eligible, examined for eligibility, confirmed eligible, included in the study, completing follow-up, and analysed | Fig S1 |
|  |  | (b) Give reasons for non-participation at each stage |  |
|  |  | (c) Consider use of a flow diagram |  |
| Descriptive data | 14* | (a) Give characteristics of study participants (eg demographic, clinical, social) and information on exposures and potential confounders | Table 1 |
|  |  | (b) Indicate number of participants with missing data for each variable of interest | Fig S1 |
|  |  | (c) Summarise follow-up time (eg, average and total amount) |  |
| Outcome data | 15* | Report numbers of outcome events or summary measures over time | Table 1 |

| Main results | 16 | (*a*) Give unadjusted estimates and, if applicable, confounder-adjusted estimates and their precision (eg, 95% confidence interval). Make clear which confounders were adjusted for and why they were included | Table S2 |
| --- | --- | --- | --- |
|  |  | (*b*) Report category boundaries when continuous variables were categorized |  |
|  |  | (*c*) If relevant, consider translating estimates of relative risk into absolute risk for a meaningful time period |  |
| Other analyses | 17 | Report other analyses done—eg analyses of subgroups and interactions, and sensitivity analyses | Supp Methods |
| Discussion | | | |
| Key results | 18 | Summarise key results with reference to study objectives | 10 |
| Limitations | 19 | Discuss limitations of the study, taking into account sources of potential bias or imprecision. Discuss both direction and magnitude of any potential bias | 12 |
| Interpretation | 20 | Give a cautious overall interpretation of results considering objectives, limitations, multiplicity of analyses, results from similar studies, and other relevant evidence | 13 |
| Generalisability | 21 | Discuss the generalisability (external validity) of the study results | 12 |
| Other information | | | |
| Funding | 22 | Give the source of funding and the role of the funders for the present study and, if applicable, for the original study on which the present article is based | 15 |

*Give information separately for exposed and unexposed groups.

**Note:** An Explanation and Elaboration article discusses each checklist item and gives methodological background and published examples of transparent reporting. The STROBE checklist is best used in conjunction with this article (freely available on the Web sites of PLoS Medicine at http://www.plosmedicine.org/, Annals of Internal Medicine at http://www.annals.org/, and Epidemiology at http://www.epidem.com/). Information on the STROBE Initiative is available at http://www.strobe-statement.org.

### **Figure S1.** Flow Chart of the Study Population Examining Factors Associated with Fall Related Injury Events among Incident and Continuing Benzodiazepine Users.

**B. Continuing Cohort**

**A. Incident Cohort**

**744,153** with at least one BZD where 65 years or older on day filled

**3,994,494** excluded

**3,994,494** no BZD use between 4/1/16 and 12/31/17 during 6+ month period of continuous coverage

**5,170,845** alive on 4/1/16, resided in 50 states/DC, with 6+ months continuous fee-for-service and Part D coverage between 10/1/15 and 12/31/17^a^

**1,176,351** with at least one BZD filled between 4/1/16 and 12/31/17 during 6+ month period of continuous coverage

**146,436** excluded

**146,436** no BZD fill preceded by 6 months of continuous coverage

**1,029,915** with at least one BZD fill preceded by 6 months continuous coverage

**744,153** with at least one BZD where 65 years or older on day filled

**285,762** excluded

**285,762** < 65 years old on days BZDs that met previous criteria filled

**16,669** had an OD event in the previous 6 months

**205,529** excluded

**205,529** no BZDs filled in prior 6 months

**538,624** at least one BZD fill and 1+ BZD fill in prior 6 months^b^

**5,170,845** alive on 4/1/16, resided in 50 states/DC, with 6+ months continuous fee-for-service and Part D coverage between 10/1/15 and 12/31/17^a^

**3,994,494** excluded

**3,994,494** no BZD use between 4/1/16 and 12/31/17 during 6+ month period of continuous coverage

**146,436** excluded

**146,436** no BZD fill preceded by 6 months of continuous coverage

**1,176,351** with at least one BZD filled between 4/1/16 and 12/31/17 during 6+ month period of continuous coverage

**21,630** excluded

**89** died before day of Continuing BZD use

**21,541** had fall related event in prior 6 months

**516,994** alive on day of continuing BZD use, no fall related events in prior 6 months

**7,360** excluded

**7,360** missing race, rurality, or census division

**509,634** continuing BZD users with no missing data

**379,273** incident BZD users with no missing data

**385,708** alive on day of incident BZD use, no fall related events in prior 6 months

**6,435** excluded

**6,435** missing race, rurality, or census division

**17,489** excluded

**62** died before day of incident BZD use

**17,427** had fall related event in prior 6 months

**403,197** at least one BZD fill and no BZD fill in prior 6 months^b^

**340,956** excluded

**340,956** BZDs filled in previous 6 months

**285,762** excluded

**285,762** < 65 years old on days BZDs that met previous criteria filled

**16,669** had an OD event in the previous 6 months

**1,029,915** with at least one BZD fill preceded by 6 months continuous coverage

## BZD, benzodiazepine; OD, overdose

## ^a^ Among a Medicare 20% sample. Continuous fee for-service means that a given beneficiary had continuous Parts A and B and no Part C coverage. Part A is hospital insurance and covers hospital stays including inpatient and skilled nursing stays. Part B is medical insurance covers services and equipment such as office visits and wheelchairs. Part C (or Medicare Advantage) means that a given beneficiary elects to obtain their Part A and B insurance coverage through a private health insurance company; those with coverage not through a private health insurance company are considered “fee-for-service” (or “traditional Medicare”). Part D is prescription drug coverage.

^b^ If multiple BZD fills met the inclusion criteria for a given beneficiary, the earliest was selected. The BZD filled (incident or continuing) is referred to as the index BZD prescription.

## **Table S1.** Medications Contributing to Each Medication Class

| **Medication class** | **Source** | **Medications** |
| --- | --- | --- |
| Benzodiazepines | AHFS: 28:24.08 | Alprazolam, Chlordiazepoxide, Clorazepate, Diazepam, Estazolam, Flurazepam, Halazepam, Lorazepam, Midazolam, Oxazepam, Prazepam, Quazepam, Temazepam, Triazolam, Clobazam, Clonazepam |
| Antidepressants | AHFS: 28:16.04.xx | Isocarboxazid, Phenelzine, Tranylcypromine, Rasagiline, SelegilineDesvenlafaxine, Duloxetine, Levomilnacipran,Venlafaxine, MilnacipranCitalopram, Escitalopram, Fluoxetine, Fluvoxamine, Paroxetine, Sertraline, Vilazodone, Nefazodone, Trazodone, Vortioxetine, Amitriptyline, Amoxapine, Clomipramine, Desipramine, Doxepin, Imipramine, Maprotiline, Nortriptyline, Protriptyline, Trimipramine, Bupropion, Mirtazapine |
| Antiepileptics | AHFS:except benzodiazepines | Phenobarbital, Primidone, Methohexital, Ethotoin, Fosphenytoin, PhenytoinEthadione, Paramethadione, Trimethadione, Ethosuximide, MethsuximideBrivaracetam, Carbamazepine, Eslicarbazepine, Felbamate, Gabapentin, Lacosamide, Lamotrigine, Levetiracetam, Magnesium Sulfate, Oxcarbazepine, Perampanel, Pregabalin, Rufinamide, Sultiame, Tiagabine, Topiramate, Valproate/Divalproex/Valproic Acid, Vigabatrin, Zonisamide, Acetazolamide |
| Antipsychotics | AHFS: 28:16.08.xx | Aripiprazole, Asenapine, Brexpiprazole, Cariprazine, Clozapine, Iloperidone, Lurasidone, Olanzapine, Paliperidone, Pimavanserin, Quetiapine, Risperidone, Ziprasidone, Haloperidol, Chlorpromazine, Fluphenazine, Perphenazine, Prochlorperazine, Thioridazine, TrifluoperazineThiothixene,Loxapine, Molindone, Pimozide |
| Opioids | AHFS: 28:08.08 and 28:08.12 | Codeine, Fentanyl, Hydrocodone, Hydromorphone Levorphanol, Meperidine, Methadone, Morphine, Opium, Oxycodone, Oxymorphone, Remifentanil, Sufentanil, Tapentadol, Tramadol, Buprenorphine, Butorphanol, Nalbuphine, Pentazocine, Dihydrocodeine |
| Z-drugs | AHFS: Subset of 28:24.92 | Eszopiclone, Zaleplon, Zolpidem |
| Antihypertensives | Sussman et al.^[[1]](#footnote-2)^ | Acebutolol, Atenolol, Betaxolol, Bisoprolol, Carvedilol, Labetalol, Metoprolol, Nadolol, Nebivolol, Penbutolol, Pindolol, Propranolol, Timolol, Doxazosin, Prazosin, Terazosin, Amlodipine, Diltiazem, Felodipine, Isradipine, Nicardipine, Nifedipine, Verapamil, Clonidine, Guanabenz, Guanfacine, Hydralazine, Methyldopa, Minoxidil, Chlorothiazide, Chlorthalidone, Hydrochlorothiazide, Indapamide, Metolazone, Bumetanide, Furosemide, Torsemide, Amiloride, Eplerenone, Spironolactone, Spironolactone, Benazepril, Captopril, Enalapril, Fosinopril, Lisinopril, Moexipril, Perindopril, Quinapril, Ramipril, Trandolapril, Candesartan, Eprosartan, Irbesartan, Losartan, Olmesartan, Telmisartan, Valsartan, Aliskiren |
| Parkinson’s medications | Connolly et al.^[[2]](#footnote-3)^ | Levodopa-carbidopa, Levodopa-benserazide, Pramipexole, Ropinirole, Rotigotine, Selegiline, Rasagiline, Entacapone, Tolcapone, Amantadine, Trihexyphenidyl, Benztropine |

AHFS, American Hospital Formulary Service; VA, Veterans Affairs.

**Figure S2.** Computing Medication Possession Ratio (MPR) for Continuing Benzodiazepine Users^a^


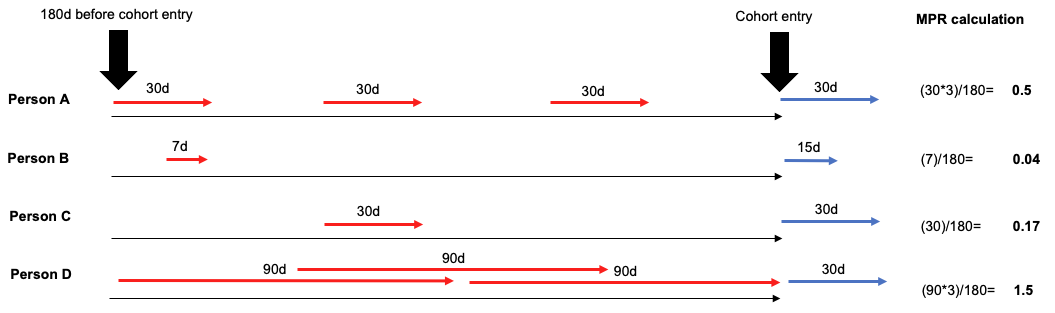


MPR, medication possession ratio; d, days’ supply.

^a^ Time of cohort entry for each person is indicated by the blue arrows (i.e., index BZD fill). Red arrows indicate BZD prescription fills during the 6-month baseline prior to cohort entry. MPR was calculated based by summing these baseline BZD fill dates and dividing by 180.

## **Table S2.** Characteristics Associated with Fall Related Injury Events Among Incident and Continuing Benzodiazepine Users^a^

|  | **HR (95% CI)** | | | |
| --- | --- | --- | --- | --- |
|  | **Incident** | | **Continuing** | |
|  | **Unadjusted** | **Adjusted** | **Unadjusted** | **Adjusted** |
| *Sociodemographics* | | | | |
| Sex |  |  |  |  |
| Male | 1 (ref) | 1 (ref) | 1 (ref) | 1 (ref) |
| Female | 0.94 (0.83-1.06) | 0.90 (0.80-1.02) | 1.21 (1.12-1.31)*** | 1.09 (1.01-1.18)* |
| Age |  |  |  |  |
| 65-74 | 1 (ref) | 1 (ref) | 1 (ref) | 1 (ref) |
| 75-84 | 2.15 (1.94-2.38)*** | 1.49 (1.34-1.66)*** | 1.69 (1.56-1.84)*** | 1.40 (1.29-1.53)*** |
| 85+ | 4.65 (4.07-5.31)*** | 2.08 (1.81-2.39)*** | 3.30 (3.02-3.60)*** | 2.14 (1.94-2.36)*** |
| Race^b^ |  |  |  |  |
| Non-Hispanic White | 1 (ref) | 1 (ref) | 1 (ref) | 1 (ref) |
| Non-Hispanic Black | 1.03 (0.83-1.28) | 0.84 (0.68-1.04) | 1.05 (0.89-1.24) | 0.86 (0.73-1.02) |
| Hispanic | 0.90 (0.76-1.06) | 0.86 (0.72-1.03) | 0.84 (0.71-0.99)* | 0.74 (0.63-0.88)*** |
| Asian/Pacific Islander | 0.88 (0.59-1.31) | 1.14 (0.76-1.71) | 0.75 (0.55-1.04) | 0.83 (0.60-1.15) |
| Other | 1.22 (0.38-3.90) | 1.21 (0.42-3.53) | 1.14 (0.81-1.60) | 1.13 (0.81-1.60) |
| Low-income subsidy^c^ |  |  |  |  |
| No | 1 (ref) | 1 (ref) | 1 (ref) | 1 (ref) |
| Yes | 1.73 (1.55-1.93)*** | 0.81 (0.71-0.91)*** | 1.60 (1.49-1.72)*** | 0.92 (0.85-0.99)* |
| Rurality^d^ |  |  |  |  |
| Urban | 1 (ref) | 1 (ref) | 1 (ref) | 1 (ref) |
| Rural | 1.19 (1.01-1.39)* | 1.00 (0.85-1.17) | 1.04 (0.93-1.16) | 0.93 (0.83-1.04) |
| *Clinical Characteristics^e^* |  |  |  |  |
| Elixhauser^f^ |  |  |  |  |
| 0-1 | 1 (ref) | 1 (ref) | 1 (ref) | 1 (ref) |
| 2 | 1.40 (1.21-1.61)*** | 0.84 (0.72-0.97)* | 1.42 (1.24-1.63)*** | 0.94 (0.81-1.08) |
| 3 | 1.83 (1.58-2.11)*** | 0.76 (0.65-0.89)*** | 1.92 (1.69-2.19)*** | 0.92 (0.80-1.07) |
| 4 | 2.48 (2.13-2.88)*** | 0.80 (0.67-0.95)* | 2.25 (1.97-2.58)*** | 0.83 (0.71-0.97)* |
| 5 | 2.88 (2.48-3.34)*** | 0.74 (0.62-0.87)*** | 2.85 (2.49-3.27)*** | 0.87 (0.74-1.02) |
| 6 | 3.78 (3.12-4.59)*** | 0.81 (0.66-1.00) | 3.18 (2.72-3.72)*** | 0.84 (0.70-1.00) |
| 7 | 4.61 (3.67-5.78)*** | 0.89 (0.69-1.15) | 3.82 (3.28-4.47)*** | 0.91 (0.76-1.09) |
| 8 | 4.84 (4.06-5.77)*** | 0.82 (0.67-1.01) | 4.48 (3.76-5.33)*** | 0.97 (0.80-1.19) |
| 9+ | 6.91 (5.65-8.46)*** | 1.06 (0.88-1.27) | 6.13 (5.31-7.06)*** | 1.26 (1.06-1.50)** |
| Dementia | 4.41 (3.93-4.96)*** | 1.27 (1.12-1.45)*** | 3.46 (3.20-3.74)*** | 1.10 (1.00-1.21) |
| Osteoarthritis | 1.56 (1.41-1.73)*** | 1.03 (0.92-1.15) | 1.64 (1.53-1.76)*** | 1.07 (0.99-1.15) |
| Stroke | 2.39 (1.92-2.98)*** | 1.10 (0.92-1.33) | 1.98 (1.76-2.22)*** | 1.03 (0.91-1.16) |
| Substance Use Disorder | 1.30 (1.15-1.47)*** | 0.98 (0.86-1.12) | 1.44 (1.31-1.59)*** | 1.14 (1.02-1.27)* |
| Urinary incontinence­­­ | 1.72 (1.48-2.00)*** | 1.03 (0.88-1.21) | 1.73 (1.54-1.95)*** | 1.09 (0.96-1.23) |
| Antihypertensive medication | 1.64 (1.47-1.83)*** | 1.05 (0.93-1.18) | 1.38 (1.26-1.50)*** | 0.92 (0.84-1.01) |
| Parkinson’s disease medication | 1.69 (1.45-1.96)*** | 0.98 (0.84-1.14) | 1.58 (1.41-1.77)*** | 0.95 (0.85-1.07) |
| Frailty | 10.11 (9.14-11.18)*** | 7.03 (6.16-8.03)*** | 7.78 (7.14-8.48)*** | 5.72 (5.07-6.46)*** |
| *BZD Characteristics* | | | | |
| Medication possession ratio |  |  |  |  |
| <0.5 |  |  |  |  |
| Day 1-10 | - | - | 1.38 (1.23, 1.56)*** | 1.23 (1.08, 1.39)** |
| Day 11-20 | - | - | 1.01 (0.89, 1.15) | 0.93 (0.81, 1.07) |
| Day 21-30 | - | - | 1.04 (0.91, 1.19) | 0.99 (0.86, 1.14) |
| 0.5-1 | - | - | 1 (ref) | 1 (ref) |
| >1 |  |  |  |  |
| Day 1-10 | - | - | 1.48 (1.13, 1.94)** | 1.16 (0.88, 1.51) |
| Day 11-20 | - | - | 0.94 (0.66, 1.34) | 0.73 (0.51, 1.03) |
| Day 21-30 | - | - | 1.43 (1.06, 1.94)* | 1.15 (0.85, 1.55) |
| Avg daily dose in lor-eq of index BZD |  |  |  |  |
| <1 | 1 (ref) | 1 (ref) | 1 (ref) | 1 (ref) |
| 1-1.99 | 1.00 (0.91-1.11) | 0.97 (0.87-1.07) | 1.00 (0.91-1.09) | 1.04 (0.95-1.14) |
| 2+ | 1.22 (1.06-1.41)** | 1.02 (0.90-1.16) | 0.96 (0.88-1.05) | 1.09 (0.99-1.20) |
| Days’ supply of index BZD |  |  |  |  |
| <14 |  |  |  |  |
| Day 1-10 | 1.45 (1.26, 1.68)*** | 1.37 (1.19, 1.59)*** | 1.81 (1.53, 2.14)*** | 1.26 (1.07, 1.48)** |
| Day 11-20 | 1.07 (0.84, 1.37) | 1.02 (0.81, 1.29) | 1.48 (1.24, 1.76)*** | 1.11 (0.92, 1.33) |
| Day 21-30 | 0.90 (0.73, 1.12) | 0.86 (0.70, 1.06) | 1.20 (0.99, 1.44) | 0.89 (0.73, 1.08) |
| 14-30 | 1 (ref) | 1 (ref) | 1 (ref) | 1 (ref) |
| 31+ |  |  |  |  |
| Day 1-10 | 0.52 (0.41, 0.67)*** | 0.70 (0.54, 0.91)** | 0.66 (0.56, 0.78)*** | 0.85 (0.72, 1.02) |
| Day 11-20 | 0.54 (0.41, 0.72)*** | 0.72 (0.55, 0.97)* | 0.72 (0.60, 0.88)*** | 0.95 (0.78, 1.15) |
| Day 21-30 | 0.67 (0.50, 0.89)*** | 0.90 (0.67, 1.20) | 0.56 (0.45, 0.70)*** | 0.73 (0.58, 0.90)** |
| *Other Medication Use^g^* | | | | |
| Antidepressants |  |  |  |  |
| Former | 1.67 (1.36-2.05)*** | 0.97 (0.78-1.20) | 1.54 (1.35-1.75)*** | 1.10 (0.96-1.25) |
| Current | 1.74 (1.57-1.93)*** | 1.09 (0.98-1.21) | 1.89 (1.75-2.03)*** | 1.30 (1.20-1.40)*** |
| Antiepileptics |  |  |  |  |
| Former | 1.96 (1.43-2.68)*** | 1.30 (0.98-1.71) | 1.47 (1.29-1.68)*** | 1.06 (0.93-1.21) |
| Current | 1.72 (1.54-1.91)*** | 1.07 (0.96-1.19) | 1.76 (1.63-1.91)*** | 1.16 (1.06-1.26)*** |
| Antipsychotics |  |  |  |  |
| Former | 2.68 (1.98-3.62)*** | 1.34 (0.99-1.80) | 2.25 (1.94-2.60)*** | 1.34 (1.15-1.56)*** |
| Current | 2.50 (2.17-2.88)*** | 1.21 (1.03-1.40)* | 2.13 (1.93-2.35)*** | 1.23 (1.10-1.37)*** |
| Opioids |  |  |  |  |
| Former | 1.33 (1.20-1.47)*** | 1.07 (0.96-1.20) | 1.26 (1.15-1.37)*** | 0.99 (0.91-1.09) |
| Current | 1.84 (1.60-2.12)*** | 1.22 (1.07-1.40)** | 1.66 (1.53-1.80)*** | 1.21 (1.11-1.33)*** |
| Z-drugs |  |  |  |  |
| Former | 0.55 (0.43-0.71)*** | 0.63 (0.49-0.81)*** | 0.90 (0.75-1.08) | 0.96 (0.80-1.15) |
| Current | 0.72 (0.59-0.89)** | 0.94 (0.76-1.16) | 0.88 (0.74-1.03) | 1.03 (0.87-1.22) |

*p<.05; **<.01; ***<.001

HR, hazard ratio; CI, confidence interval; BZD, benzodiazepine; lor-eq, lorazepam-equivalent.

^a^ Adjusted models additionally accounted for census division, seasonality (month of index BZD prescription), and their interaction.

^b^ Derived using the Research Triangle Institute race variable; race groups are mutually exclusive.

^c^ Considered present if a given beneficiary was enrolled or eligible in the Part D low-income subsidy for at least one month during the 6-month baseline period.

^d^ Derived using beneficiary state and county codes and Rural-Urban Continuum Codes.

^e^ The reference group for all categories is patients in whom the row condition is not present, with the exception of Elixhauser.

^f^ Excludes depression and substance use disorders, which were captured separately.

^g^ The reference group for all medication exposures is never use.

**Table S3.** Distribution of Characteristics of Incident and Continuing Benzodiazepine Users by Days’ Supply: Before Weighting

|  | **N (%)** | | | | | |
| --- | --- | --- | --- | --- | --- | --- |
|  | **Incident BZD Cohort**  **N=379,273** | | | **Continuing BZD Cohort**  **N=509,634** | | |
|  | **<14 days’ supply**  **N=144,816** | **14-30 days’ supply**  **N=201,315** | **>30 days’ supply**  **N=33,142** | **<14 days’ supply**  **N=59,415** | **14-30 days’ supply**  **N=371,159** | **>30 days’ supply**  **N=79,060** |
| *Sociodemographics* |  |  |  |  |  |  |
| Sex |  |  |  |  |  |  |
| Male | 47,159 (32.6) | 60,385 (30.0) | 10,492 (31.7) | 17,174 (28.9) | 106,708 (28.7) | 25,560 (32.3) |
| Female | 97,657 (67.4) | 140,930 (70.0) | 22,650 (68.3) | 42,241 (71.1) | 264,451 (71.3) | 53,500 (67.7) |
| Age |  |  |  |  |  |  |
| 65-74 | 80,732 (55.7) | 104,408 (51.9) | 17,526 (52.9) | 31,814 (53.5) | 208,038 (56.1) | 43,621 (55.2) |
| 75-84 | 43,275 (29.9) | 65,294 (32.4) | 11,386 (34.4) | 17,264 (29.1) | 110,462 (29.8) | 25,544 (32.3) |
| 85+ | 20,809 (14.4) | 31,613 (15.7) | 4,230 (12.8) | 10,337 (17.4) | 52,659 (14.2) | 9,895 (12.5) |
| Race |  |  |  |  |  |  |
| Non-Hispanic White | 127,973 (88.4) | 172,768 (85.8) | 30,160 (91.0) | 52,812 (88.9) | 322,240 (86.8) | 72,585 (91.8) |
| Non-Hispanic Black | 7,668 (5.3) | 10,174 (5.1) | 941 (2.8) | 3,113 (5.2) | 19,121 (5.2) | 1,963 (2.5) |
| Hispanic | 5,920 (4.1) | 11,718 (5.8) | 1,243 (3.8) | 2,274 (3.8) | 21,143 (5.7) | 3,064 (3.9) |
| Asian/Pacific Islander | 2,003 (1.4) | 4,888 (2.4) | 555 (1.7) | 681 (1.1) | 5,649 (1.5) | 930 (1.2) |
| Other | 1,252 (0.9) | 1,767 (0.9) | 243 (0.7) | 535 (0.9) | 3,006 (0.8) | 518 (0.7) |
| Low-income subsidy |  |  |  |  |  |  |
| No | 111,862 (77.2) | 153,282 (76.1) | 29,710 (89.6) | 40,436 (68.1) | 252,739 (68.1) | 68,383 (86.5) |
| Yes | 32,954 (22.8) | 48,033 (23.9) | 3,432 (10.4) | 18,979 (31.9) | 118,420 (31.9) | 10,677 (13.5) |
| Rurality |  |  |  |  |  |  |
| Urban | 125,456 (86.6) | 176,056 (87.5) | 29,679 (89.6) | 50,920 (85.7) | 316,202 (85.2) | 70,099 (88.7) |
| Rural | 19,360 (13.4) | 25,259 (12.5) | 3,463 (10.4) | 8,495 (14.3) | 54,957 (14.8) | 8,961 (11.3) |
| *Clinical Characteristics* |  |  |  |  |  |  |
| Elixhauser |  |  |  |  |  |  |
| 0-1 | 39,120 (27.0) | 55,222 (27.4) | 10,509 (31.7) | 13,350 (22.5) | 94,760 (25.5) | 23,610 (29.9) |
| 2 | 24,835 (17.1) | 35,079 (17.4) | 6,597 (19.9) | 9,486 (16.0) | 68,609 (18.5) | 16,002 (20.2) |
| 3 | 21,882 (15.1) | 30,292 (15.0) | 5,210 (15.7) | 9,144 (15.4) | 59,358 (16.0) | 13,163 (16.6) |
| 4 | 17,111 (11.8) | 23,293 (11.6) | 3,790 (11.4) | 7,389 (12.4) | 45,820 (12.3) | 9,450 (12.0) |
| 5 | 12,810 (8.8) | 17,212 (8.5) | 2,432 (7.3) | 5,532 (9.3) | 33,652 (9.1) | 6,176 (7.8) |
| 6 | 8,960 (6.2) | 12,338 (6.1) | 1,635 (4.9) | 4,356 (7.3) | 23,190 (6.2) | 4,032 (5.1) |
| 7 | 6,443 (4.4) | 8,738 (4.3) | 1,070 (3.2) | 3,172 (5.3) | 15,718 (4.2) | 2,581 (3.3) |
| 8 | 4,507 (3.1) | 6,397 (3.2) | 714 (2.2) | 2,349 (4.0) | 10,680 (2.9) | 1,569 (2.0) |
| 9+ | 9,148 (6.3) | 12,744 (6.3) | 1,185 (3.6) | 4,637 (7.8) | 19,372 (5.2) | 2,477 (3.1) |
| Dementia | 20,019 (13.8) | 24,882 (12.4) | 1,833 (5.5) | 12,666 (21.3) | 43,278 (11.7) | 4,274 (5.4) |
| Osteoarthritis | 43,671 (30.2) | 59,340 (29.5) | 9,085 (27.4) | 19,148 (32.2) | 114,775 (30.9) | 22,428 (28.4) |
| Stroke | 9,351 (6.5) | 12,115 (6.0) | 1,520 (4.6) | 4,370 (7.4) | 20,129 (5.4) | 3,278 (4.1) |
| Substance Use Disorder | 11,282 (7.8) | 15,312 (7.6) | 1,759 (5.3) | 5,780 (9.7) | 38,549 (10.4) | 5,169 (6.5) |
| Urinary incontinence | 20,019 (13.8) | 24,882 (12.4) | 1,833 (5.5) | 3,837 (6.5) | 20,571 (5.5) | 3,937 (5.0) |
| Antihypertensive | 106,174 (73.3) | 150,195 (74.6) | 24,591 (74.2) | 44,792 (75.4) | 285,957 (77.0) | 60,671 (76.7) |
| Parkinson’s disease medication | 6,538 (4.5) | 10,383 (5.2) | 1,661 (5.0) | 3,741 (6.3) | 23,893 (6.4) | 4,818 (6.1) |
| Frailty |  |  |  |  |  |  |
| Not frail | 93,907 (64.8) | 130,721 (64.9) | 25,077 (75.7) | 32,611 (54.9) | 233,379 (62.9) | 58,261 (73.7) |
| Frail | 50,909 (35.2) | 70,594 (35.1) | 8,065 (24.3) | 26,804 (45.1) | 137,780 (37.1) | 20,799 (26.3) |
| *BZD Characteristics* |  |  |  |  |  |  |
| Medication possession ratio, % |  |  |  |  |  |  |
| <0.5 | n/a | n/a | n/a | 54,923 (92.4) | 219,454 (59.1) | 45,799 (57.9) |
| 0.5-1 | n/a | n/a | n/a | 3,835 (6.5) | 140,532 (37.9) | 28,399 (35.9) |
| >1 | n/a | n/a | n/a | 657 (1.1) | 11,173 (3.0) | 4,862 (6.1) |
| Average daily dose, lor-eq mg/day |  |  |  |  |  |  |
| <1 | 28,467 (19.7) | 70,530 (35.0) | 11,984 (36.2) | 8,348 (14.1) | 82,034 (22.1) | 19,172 (24.2) |
| 1-1.99 | 70,228 (48.5) | 82,751 (41.1) | 12,146 (36.6) | 27,982 (47.1) | 139,152 (37.5) | 29,426 (37.2) |
| 2+ | 46,121 (31.8) | 48,034 (23.9) | 9,012 (27.2) | 23,085 (38.9) | 149,973 (40.4) | 30,462 (38.5) |
| *Other Medication Use* |  |  |  |  |  |  |
| Antidepressant |  |  |  |  |  |  |
| Never | 91,389 (63.1) | 114,317 (56.8) | 19,095 (57.6) | 29,458 (49.6) | 173,577 (46.8) | 40,002 (50.6) |
| Former | 9,481 (6.5) | 14,344 (7.1) | 2,003 (6.0) | 4,958 (8.3) | 32,045 (8.6) | 5,302 (6.7) |
| Current | 43,946 (30.3) | 72,654 (36.1) | 12,044 (36.3) | 24,999 (42.1) | 165,537 (44.6) | 33,756 (42.7) |
| Antiepileptics |  |  |  |  |  |  |
| Never | 114,508 (79.1) | 160,967 (80.0) | 27,394 (82.7) | 43,473 (73.2) | 279,094 (75.2) | 62,783 (79.4) |
| Former | 7,872 (5.4) | 11,210 (5.6) | 1,435 (4.3) | 3,895 (6.6) | 23,272 (6.3) | 3,764 (4.8) |
| Current | 22,436 (15.5) | 29,138 (14.5) | 4,313 (13.0) | 12,047 (20.3) | 68,793 (18.5) | 12,513 (15.8) |
| Antipsychotics |  |  |  |  |  |  |
| Never | 132,406 (91.4) | 184,232 (91.5) | 31,486 (95.0) | 49,921 (84.0) | 326,468 (88.0) | 73,230 (92.6) |
| Former | 3,301 (2.3) | 4,936 (2.5) | 442 (1.3) | 2,947 (5.0) | 11,820 (3.2) | 1,340 (1.7) |
| Current | 9,109 (6.3) | 12,147 (6.0) | 1,214 (3.7) | 6,547 (11.0) | 32,871 (8.9) | 4,490 (5.7) |
| Opioids |  |  |  |  |  |  |
| Never | 83,756 (57.8) | 130,365 (64.8) | 23,124 (69.8) | 31,178 (52.5) | 205,456 (55.4) | 50,338 (63.7) |
| Former | 25,341 (17.5) | 35,515 (17.6) | 5,621 (17.0) | 14,074 (23.7) | 77,296 (20.8) | 15,593 (19.7) |
| Current | 35,719 (24.7) | 35,435 (17.6) | 4,397 (13.3) | 14,163 (23.8) | 88,407 (23.8) | 13,129 (16.6) |
| Z-drugs |  |  |  |  |  |  |
| Never | 136,488 (94.2) | 185,193 (92.0) | 30,548 (92.2) | 54,599 (91.9) | 336,796 (90.7) | 72,954 (92.3) |
| Former | 3,289 (2.3) | 6,844 (3.4) | 868 (2.6) | 2,029 (3.4) | 15,160 (4.1) | 2,265 (2.9) |
| Current | 5,039 (3.5) | 9,278 (4.6) | 1,726 (5.2) | 2,787 (4.7) | 19,203 (5.2) | 3,841 (4.9) |

## **Supplementary Methods.** Examining the Association Between Days’ Supply and Fall-Related Injury Among Older Adults at Risk of Incident Fall-related Injury: Accounting for Imbalance in Characteristics Across Days’ Supply

Generalized propensity scores were used to address imbalance in characteristics across categories of days’ supply (i.e., <14, 14-30, >30 days’ supply; **Table S3**). Propensity scores were computed using generalized boosted models with the mnps function and twang package in R.^[[3]](#footnote-4)^ Propensity scores were then used to construct inverse probability of treatment weights with the aim of estimating the average treatment effect. For our project, days’ supply was considered the treatment and there were 3 groups (<14 days’ supply, 14-30 days’ supply, and >30 days’ supply). This means that, after applying inverse probability of treatment weights, each treatment group represents the overall cohort (e.g., incident benzodiazepine users), and the average treatment effect estimates the average effect on the outcome (fall-related injury) had everyone in the cohort received treatment 1 (days’ supply <14) vs treatment 2 (days’ supply 14-30) vs treatment 3 (days’ supply > 30).

We completed several checks as we estimated propensity scores. First, because generalized boosted models were used to estimate propensity scores, we ensured that we let the regression tree models run for enough iterations by plotting the number of iterations by the maximum of the absolute standardized mean difference (ASMD) across all characteristics in the propensity score model for each unique comparison of treatment groups (e.g., comparing treatment 1 vs 2) and ensuring that the ASMD was not still decreasing at the end of the number of iterations. Second, propensity scores were plotted by treatment group to ensure sufficient overlap in propensity scores across treatment groups and that each person had a non-zero probability of receiving each treatment. Third, we examined if balance was met for each unique pair of treatment groups (e.g., comparing dementia prevalence in treatment group 1 vs treatment group 2) by computing the ASMD for each characteristic between each unique pair of treatment groups (e.g., for dementia, we computed the ASMD comparing dementia prevalence across treatment groups 1 and 2, 1 and 3, and 2 and 3). Similar to previous research, we considered an ASMD of >0.1 to indicate imbalance.^[[4]](#footnote-5)^

Following sufficient estimation of inverse probability of treatment weights, we created a final weight that accounts for imbalance in characteristics across treatment groups and dropout. Specifically, we multiplied the inverse probability of treatment weights by the inverse probability of dropout weights, per Hernan and Robins.^[[5]](#footnote-6)^ After accounting for both imbalance in characteristics across treatment groups and dropout, each treatment group represents the overall cohort without selection bias due to dropout (**Tables S4 and S5**). This weight was then applied when estimating the relationship between days’ supply and fall-related injury with a Cox proportional hazards model (**Table S6**). We first estimated the marginal association between days’ supply and fall-related injury (i.e., examined relationship between days’ supply and fall-related injury and did not additional adjust for confounders). We then estimated the conditional association by examining the relationship between days’ supply and fall-related injury, additionally adjusting for confounders. These steps were completed separately for both the incident and continuing benzodiazepine cohorts. Finally, we trimmed final weights that were greater than the 99^th^ percentile and re-estimated conditional associations between days’ supply and fall-related injury.

Analyses were conducted using R version 4.1.0. All tests were two-sided and alpha was set at 0.05.

**Supplementary Results:** Examining the Association Between Days’ Supply and Fall-Related Injury Among Older Adults at Risk of Incident Fall-related Injury: Accounting for Imbalance in Characteristics Across Days’ Supply

Checks during propensity score estimation for both incident and continuing benzodiazepine cohorts suggested that enough iterations had been run, there was sufficient overlap in propensity scores across treatment groups, and each person in each cohort had a non-zero probability of receiving each treatment (i.e., days’ supply). ASMDs comparing characteristics between each unique combination of treatment groups (e.g., treatment group 1 and 2) were all less than 0.1, suggesting sufficient balance (**Tables S4** and **S5**).

After applying the final weights that accounted for both imbalance in characteristics across days’ supply as well as dropout, we observed differential risk of fall-related injury by day’ supply. Specifically, among the incident cohort and estimating marginal effects, relative to everyone receiving 14-30 days’ supply and preserving the population pre-dropout, days’ supply <14 was associated with an increased risk of fall-related injury during days 1-10 (**Table S6**; hazard ratio [HR] 1.42, 95% confidence interval [CI] 1.26-1.63) but not during days 11-20 or 21-30. Relative to days’ supply 14-30, days’ supply >30 was not associated with fall-related injury. Results were the same when we additionally controlled for all confounders (i.e., conditional estimates of days’ supply).

Among the continuing cohort and estimating marginal effects, relative to receiving 14-30 days’ supply, receiving <14 days’ supply was associated with an increased risk of fall-related injury during days 1-10 and 11-20 (HR 1.45, CI 1.18-1.78; and 1.39, CI 1.07-1.83, respectively) but not during days 21-30. Relative to receiving 14-30 days’ supply, receiving >30 days was associated with decreased risk of fall-related injury during days’ 21-30 (HR 0.76, CI 0.58-0.99). When we additionally controlled for confounders, days’ supply < 14 days was only associated with increased risk of fall-related injury during days 1-10; we no longer observed a relationship between days’ supply > 30 and fall-related injury.

Results did not substantively change when re-estimated conditional associations between days’ supply and fall-related injury after trimming the final weights at the 99^th^ percentile (**Table S6**).

## **Table S4.** Distribution of Characteristics of Incident Benzodiazepine Users by Days’ Supply: After Inverse Probability of Treatment Weighting^a^

|  | **N (%)** | | | **ASMD** | | |
| --- | --- | --- | --- | --- | --- | --- |
|  | **<14 days’ supply**  **N=375,974** | **14-30 days’ supply**  **N=378,149** | **>30 days’ supply**  **N=369,975** | **<14 vs.**  **14-30 days’ supply** | **<14 vs.**  **>30 days’ supply** | **14-30 vs.**  **>30 days’ supply** |
| *Sociodemographics* |  |  |  |  |  |  |
| Sex |  |  |  |  |  |  |
| Female | 258,705 (68.8) | 260,954 (69.0) | 254,947 (68.9) | 0.004 | 0.002 | 0.002 |
| Age |  |  |  |  |  |  |
| 65-74 | 202,312 (53.8) | 201,847 (53.4) | 197,167 (53.3) | 0.009 | 0.010 | 0.002 |
| 75-84 | 118,555 (31.5) | 119,702 (31.7) | 120,023 (32.4) | 0.003 | 0.020 | 0.017 |
| 85+ | 55,107 (14.7) | 56,600 (15.0) | 52,784 (14.3) | 0.009 | 0.011 | 0.020 |
| Race |  |  |  |  |  |  |
| Non-Hispanic White | 328,658 (87.4) | 329,839 (87.2) | 322,110 (87.1) | 0.006 | 0.011 | 0.005 |
| Non-Hispanic Black | 18,983 (5.0) | 18,757 (5.0) | 17,287 (4.7) | 0.004 | 0.017 | 0.013 |
| Hispanic | 18,284 (4.9) | 18,829 (5.0) | 19,772 (5.3) | 0.005 | 0.022 | 0.017 |
| Asian/Pacific Islander | 6,841 (1.8) | 7,438 (2.0) | 7,798 (2.1) | 0.011 | 0.021 | 0.010 |
| Other | 3,208 (0.9) | 3,286 (0.9) | 3,007 (0.8) | 0.002 | 0.004 | 0.006 |
| Low-income subsidy |  |  |  |  |  |  |
| Yes | 83,044 (22.1) | 84,301 (22.3) | 78,610 (21.2) | 0.005 | 0.020 | 0.025 |
| Rurality |  |  |  |  |  |  |
| Rural | 47,786 (12.7) | 47,795 (12.6) | 45,561 (12.3) | 0.002 | 0.012 | 0.010 |
| *Clinical Characteristics* |  |  |  |  |  |  |
| Elixhauser |  |  |  |  |  |  |
| 0-1 | 103,225 (27.5) | 104,605 (27.7) | 104,037 (28.1) | 0.005 | 0.015 | 0.010 |
| 2 | 66,017 (17.6) | 66,318 (17.5) | 66,057 (17.9) | 0.001 | 0.008 | 0.008 |
| 3 | 57,002 (15.2) | 57,237 (15.1) | 56,045 (15.1) | 0.001 | 0.000 | 0.000 |
| 4 | 43,856 (11.7) | 43,857 (11.6) | 42,457 (11.5) | 0.002 | 0.006 | 0.004 |
| 5 | 32,518 (8.6) | 32,308 (8.5) | 31,096 (8.4) | 0.004 | 0.009 | 0.005 |
| 6 | 22,885 (6.1) | 22,912 (6.1) | 21,911 (5.9) | 0.001 | 0.007 | 0.006 |
| 7 | 16,112 (4.3) | 16,168 (4.3) | 15,268 (4.1) | 0.000 | 0.008 | 0.007 |
| 8 | 11,514 (3.1) | 11,593 (3.1) | 11,186 (3.0) | 0.000 | 0.002 | 0.002 |
| 9+ | 22,846 (6.1) | 23,152 (6.1) | 21,917 (5.9) | 0.002 | 0.006 | 0.008 |
| Dementia | 46,592 (12.4) | 46,453 (12.3) | 42,311 (11.4) | 0.003 | 0.029 | 0.026 |
| Osteoarthritis | 111,784 (29.7) | 111,617 (29.5) | 110,502 (29.9) | 0.005 | 0.003 | 0.008 |
| Stroke | 22,991 (6.1) | 22,723 (6.0) | 20,974 (5.7) | 0.004 | 0.019 | 0.014 |
| Substance Use Disorder | 27,962 (7.4) | 28,487 (7.5) | 26,834 (7.3) | 0.004 | 0.007 | 0.011 |
| Urinary incontinence | 21,168 (5.6) | 20,928 (5.5) | 21,521 (5.8) | 0.004 | 0.008 | 0.012 |
| Antihypertensive | 277,876 (73.9) | 280,417 (74.2) | 275,186 (74.4) | 0.006 | 0.011 | 0.005 |
| Parkinson’s disease medication | 18,272 (4.9) | 18,725 (5.0) | 18,565 (5.0) | 0.004 | 0.007 | 0.003 |
| Frailty |  |  |  |  |  |  |
| Frail | 128,920 (34.3) | 129,586 (34.3) | 123,475 (33.4) | 0.000 | 0.019 | 0.019 |
| *BZD Characteristics* |  |  |  |  |  |  |
| Average daily dose, lor-eq mg/day |  |  |  |  |  |  |
| <1 | 108,328 (28.8) | 110,894 (29.3) | 111,865 (30.2) | 0.011 | 0.031 | 0.020 |
| 1-1.99 | 165,074 (43.9) | 164,473 (43.5) | 158,146 (42.7) | 0.008 | 0.023 | 0.015 |
| 2+ | 102,572 (27.3) | 102,782 (27.2) | 99,963 (27.0) | 0.002 | 0.006 | 0.004 |
| *Other Medication Use* |  |  |  |  |  |  |
| Antidepressant |  |  |  |  |  |  |
| Never | 223,267 (59.4) | 223,897 (59.2) | 219,393 (59.3) | 0.004 | 0.002 | 0.002 |
| Former | 25,668 (6.8) | 25,660 (6.8) | 24,600 (6.6) | 0.002 | 0.007 | 0.005 |
| Current | 127,039 (33.8) | 128,593 (34.0) | 125,982 (34.1) | 0.005 | 0.006 | 0.001 |
| Antiepileptics |  |  |  |  |  |  |
| Never | 299,423 (79.6) | 301,914 (79.8) | 296,189 (80.1) | 0.005 | 0.010 | 0.005 |
| Former | 20,460 (5.4) | 20,689 (5.5) | 19,417 (5.2) | 0.001 | 0.009 | 0.010 |
| Current | 56,092 (14.9) | 55,546 (14.7) | 54,368 (14.7) | 0.006 | 0.006 | 0.000 |
| Antipsychotics |  |  |  |  |  |  |
| Never | 345,669 (91.9) | 347,023 (91.8) | 340,997 (92.2) | 0.006 | 0.008 | 0.015 |
| Former | 8,428 (2.2) | 8,754 (2.3) | 7,860 (2.1) | 0.005 | 0.008 | 0.013 |
| Current | 21,878 (5.8) | 22,372 (5.9) | 21,117 (5.7) | 0.004 | 0.005 | 0.009 |
| Opioids |  |  |  |  |  |  |
| Never | 234,419 (62.3) | 237,024 (62.7) | 233,666 (63.2) | 0.007 | 0.017 | 0.010 |
| Former | 66,203 (17.6) | 66,242 (17.5) | 65,341 (17.7) | 0.002 | 0.001 | 0.004 |
| Current | 75,353 (20.0) | 74,883 (19.8) | 70,968 (19.2) | 0.006 | 0.022 | 0.016 |
| Z-drugs |  |  |  |  |  |  |
| Never | 349,764 (93.0) | 351,057 (92.8) | 343,024 (92.7) | 0.008 | 0.012 | 0.005 |
| Former | 10,453 (2.8) | 11,019 (2.9) | 10,689 (2.9) | 0.008 | 0.006 | 0.001 |
| Current | 15,757 (4.2) | 16,073 (4.3) | 16,262 (4.4) | 0.003 | 0.010 | 0.007 |

ASMD, absolute standardized mean difference.

^a^ For simplicity, Ns in this table were rounded to the nearest whole number. Month and division were also balanced (results not presented).

## **Table S5.** Distribution of Characteristics of Continuing Benzodiazepine Users by Days’ Supply: After Inverse Probability of Treatment Weighting^a^

|  | **N (%)** | | | **ASMD** | | |
| --- | --- | --- | --- | --- | --- | --- |
|  | **<14 days’ supply**  **N=477,920`** | **14-30 days’ supply**  **N=507,507** | **>30 days’ supply**  **N=492,744** | **<14 vs.  14-30 days’ supply** | **<14 vs.**  **>30 days’ supply** | **14-30 vs.**  **>30 days’ supply** |
| *Sociodemographics* |  |  |  |  |  |  |
| Sex |  |  |  |  |  |  |
| Female | 341,263 (71.4) | 359,095 (70.8) | 347,809 (70.6) | 0.014 | 0.018 | 0.004 |
| Age |  |  |  |  |  |  |
| 65-74 | 266,738 (55.8) | 282,930 (55.7) | 276,808 (56.2) | 0.001 | 0.007 | 0.009 |
| 75-84 | 141,805 (29.7) | 152,146 (30.0) | 148,583 (30.2) | 0.007 | 0.011 | 0.004 |
| 85+ | 69,378 (14.5) | 72,432 (14.3) | 67,352 (13.7) | 0.007 | 0.024 | 0.017 |
| Race |  |  |  |  |  |  |
| Non-Hispanic White | 420,744 (88.0) | 445,511 (87.8) | 432,935 (87.9) | 0.008 | 0.005 | 0.002 |
| Non-Hispanic Black | 22,893 (4.8) | 24,164 (4.8) | 22,796 (4.6) | 0.001 | 0.008 | 0.006 |
| Hispanic | 24,116 (5.0) | 26,550 (5.2) | 25,938 (5.3) | 0.008 | 0.010 | 0.001 |
| Asian/Pacific Islander | 6,522 (1.4) | 7,236 (1.4) | 7,331 (1.5) | 0.005 | 0.010 | 0.005 |
| Other | 3,645 (0.8) | 4,045 (0.8) | 3,744 (0.8) | 0.004 | 0.000 | 0.004 |
| Low-income subsidy |  |  |  |  |  |  |
| Yes | 144,928 (30.3) | 147,712 (29.1) | 133,587 (27.1) | 0.027 | 0.071 | 0.044 |
| Rurality |  |  |  |  |  |  |
| Rural | 63,546 (13.3) | 72,298 (14.2) | 67,543 (13.7) | 0.027 | 0.012 | 0.015 |
| *Clinical Characteristics* |  |  |  |  |  |  |
| Elixhauser |  |  |  |  |  |  |
| 0-1 | 120,888 (25.3) | 131,338 (25.9) | 129,842 (26.4) | 0.013 | 0.024 | 0.011 |
| 2 | 86,284 (18.1) | 93,700 (18.5) | 92,791 (18.8) | 0.011 | 0.020 | 0.010 |
| 3 | 73,793 (15.4) | 81,173 (16.0) | 78,908 (16.0) | 0.015 | 0.016 | 0.001 |
| 4 | 59,132 (12.4) | 62,240 (12.3) | 59,539 (12.1) | 0.003 | 0.009 | 0.006 |
| 5 | 43,449 (9.1) | 45,314 (8.9) | 42,391 (8.6) | 0.006 | 0.017 | 0.011 |
| 6 | 30,623 (6.4) | 31,393 (6.2) | 29,948 (6.1) | 0.009 | 0.014 | 0.004 |
| 7 | 21,535 (4.5) | 21,341 (4.2) | 19,998 (4.1) | 0.015 | 0.022 | 0.007 |
| 8 | 14,960 (3.1) | 14,532 (2.9) | 13,957 (2.8) | 0.016 | 0.018 | 0.002 |
| 9+ | 27,257 (5.7) | 26,477 (5.2) | 25,370 (5.1) | 0.022 | 0.025 | 0.003 |
| Dementia | 59,943 (12.5) | 60,288 (11.9) | 54,298 (11.0) | 0.021 | 0.047 | 0.027 |
| Osteoarthritis | 149,253 (31.2) | 155,396 (30.6) | 151,663 (30.8) | 0.013 | 0.010 | 0.003 |
| Stroke | 27,924 (5.8) | 27,594 (5.4) | 26,270 (5.3) | 0.018 | 0.023 | 0.005 |
| Substance Use Disorder | 48,279 (10.1) | 49,591 (9.8) | 46,671 (9.5) | 0.011 | 0.021 | 0.010 |
| Urinary incontinence | 28,877 (6.0) | 28,051 (5.5) | 28,094 (5.7) | 0.022 | 0.015 | 0.008 |
| Antihypertensive | 363,391 (76.0) | 389,421 (76.7) | 378,525 (76.8) | 0.016 | 0.019 | 0.002 |
| Parkinson’s disease medication | 31,043 (6.5) | 32,213 (6.3) | 31,688 (6.4) | 0.006 | 0.003 | 0.003 |
| Frailty |  |  |  |  |  |  |
| Frail | 184,150 (38.5) | 185,235 (36.5) | 174,260 (35.4) | 0.042 | 0.066 | 0.024 |
| *BZD Characteristics* |  |  |  |  |  |  |
| Medication possession ratio, % |  |  |  |  |  |  |
| <0.5 | 316,435 (66.2) | 319,860 (63.0) | 311,442 (63.2) | 0.066 | 0.062 | 0.004 |
| 0.5-1 | 146,411 (30.6) | 171,209 (33.7) | 164,598 (33.4) | 0.065 | 0.059 | 0.007 |
| >1 | 15,075 (3.2) | 16,438 (3.2) | 16,704 (3.4) | 0.005 | 0.013 | 0.008 |
| Average daily dose, lor-eq mg/day |  |  |  |  |  |  |
| <1 | 98,707 (20.7) | 108,918 (21.5) | 107,218 (21.8) | 0.020 | 0.027 | 0.007 |
| 1-1.99 | 191,780 (40.1) | 195,725 (38.6) | 188,599 (38.3) | 0.032 | 0.038 | 0.006 |
| 2+ | 187,433 (39.2) | 202,864 (40.0) | 196,927 (40.0) | 0.015 | 0.015 | 0.000 |
| *Other Medication Use* |  |  |  |  |  |  |
| Antidepressant |  |  |  |  |  |  |
| Never | 219,401 (45.9) | 241,455 (47.6) | 235,206 (47.7) | 0.033 | 0.037 | 0.003 |
| Former | 41,814 (8.7) | 42,392 (8.4) | 40,264 (8.2) | 0.014 | 0.021 | 0.007 |
| Current | 216,705 (45.3) | 223,660 (44.1) | 217,274 (44.1) | 0.026 | 0.025 | 0.000 |
| Antiepileptics |  |  |  |  |  |  |
| Never | 355,150 (74.3) | 383,716 (75.6) | 374,550 (76.0) | 0.030 | 0.040 | 0.009 |
| Former | 31,410 (6.6) | 31,004 (6.1) | 29,039 (5.9) | 0.019 | 0.028 | 0.009 |
| Current | 91,360 (19.1) | 92,787 (18.3) | 89,155 (18.1) | 0.022 | 0.026 | 0.005 |
| Antipsychotics |  |  |  |  |  |  |
| Never | 417,311 (87.3) | 447,713 (88.2) | 436,591 (88.6) | 0.028 | 0.040 | 0.012 |
| Former | 17,043 (3.6) | 16,120 (3.2) | 15,101 (3.1) | 0.022 | 0.029 | 0.006 |
| Current | 43,566 (9.1) | 43,675 (8.6) | 41,051 (8.3) | 0.018 | 0.028 | 0.010 |
| Opioids |  |  |  |  |  |  |
| Never | 261,389 (54.7) | 285,725 (56.3) | 281,950 (57.2) | 0.032 | 0.051 | 0.019 |
| Former | 104,637 (21.9) | 106,333 (21.0) | 103,258 (21.0) | 0.023 | 0.023 | 0.000 |
| Current | 111,894 (23.4) | 115,449 (22.7) | 107,535 (21.8) | 0.016 | 0.038 | 0.022 |
| Z-drugs |  |  |  |  |  |  |
| Never | 432,999 (90.6) | 462,150 (91.1) | 449,015 (91.1) | 0.016 | 0.018 | 0.002 |
| Former | 19,847 (4.2) | 19,539 (3.9) | 18,560 (3.8) | 0.016 | 0.020 | 0.004 |
| Current | 25,074 (5.2) | 25,818 (5.1) | 25,168 (5.1) | 0.007 | 0.006 | 0.001 |

ASMD, absolute standardized mean difference.

^a^ For simplicity, Ns in this table were rounded to the nearest whole number. Month and division were also balanced (results not presented).

## **Table S6.** Association Between Days’ Supply and Fall-Related Injury After Accounting for Imbalance in Characteristics Across Days’ Supply and Dropout: Incident and Continuing BZD Users^a^

|  | **HR (95% CI)** | | | |
| --- | --- | --- | --- | --- |
|  | **Incident** | | **Continuing** | |
|  | **<14 vs 14-30 days’ supply** | **>30 vs 14-30 days’ supply** | **<14 vs 14-30 days’ supply** | **>30 vs 14-30 days’ supply** |
| Marginal^b^ |  |  |  |  |
| Time-dependent coefficients |  |  |  |  |
| Day 1-10 | 1.42 (1.23, 1.63) | 0.80 (0.60, 1.06) | 1.45 (1.18, 1.78) | 0.92 (0.74, 1.16) |
| Day 11-20 | 1.07 (0.87, 1.31) | 0.79 (0.58, 1.09) | 1.39 (1.07, 1.83) | 0.96 (0.76, 1.21) |
| Day 21-30 | 0.90 (0.75, 1.09) | 0.91 (0.66, 1.27) | 1.06 (0.81, 1.38) | 0.76 (0.58, 0.99) |
| Conditional^c^ |  |  |  |  |
| Time-dependent coefficients |  |  |  |  |
| Day 1-10 | 1.38 (1.20, 1.59) | 0.84 (0.63, 1.11) | 1.35 (1.09, 1.66) | 0.97 (0.77, 1.21) |
| Day 11-20 | 1.05 (0.85, 1.28) | 0.83 (0.61, 1.15) | 1.30 (0.99, 1.71) | 1.00 (0.79, 1.27) |
| Day 21-30 | 0.89 (0.73, 1.07) | 0.96 (0.69, 1.34) | 0.99 (0.76, 1.29) | 0.80 (0.61, 1.04) |
| Conditional with trimmed weights^d^ |  |  |  |  |
| Time-dependent coefficients |  |  |  |  |
| Day 1-10 | 1.31 (1.16, 1.49) | 0.78 (0.60, 1.02) | 1.31 (1.10, 1.54) | 0.93 (0.75, 1.14) |
| Day 11-20 | 1.01 (0.87, 1.18) | 0.87 (0.65, 1.18) | 1.15 (0.93, 1.42) | 1.02 (0.81, 1.27) |
| Day 21-30 | 0.87 (0.73, 1.03) | 0.99 (0.73, 1.34) | 0.98 (0.78, 1.22) | 0.80 (0.62, 1.02) |

HR, hazard ratio; CI, confidence interval.

^a^ Estimates computed from a Cox proportional hazards model weighted by a weight constructed by multiplying the inverse probability of treatment (days’ supply) weight by the dropout weight per Hernan and Robins. Logistic regression was used to create dropout weights.

^b^ Marginal estimates come from a weighted Cox proportional hazards model with only treatment included.

^c^ Conditional estimates come from a weighted Cox proportional hazards model with treatment and all confounders included to account for residual differences in confounders across treatment groups.

^d^ Conditional with trimmed weights estimates are from the same model as for Conditional, except with weights trimmed at the 99^th^ percentile prior to fitting the model.

1. Sussman JB, Kerr EA, Saini SD, Holleman RG, Klamerus ML, Min LC, *et al.* Rates of Deintensification of Blood Pressure and Glycemic Medication Treatment Based on Levels of Control and Life Expectancy in Older Patients With Diabetes Mellitus. *JAMA Intern Med* 2015;175:1942. [↑](#footnote-ref-2)
2. Connolly BS, Lang AE. Pharmacological treatment of Parkinson disease: a review. *JAMA* 2014;311:1670–83. [↑](#footnote-ref-3)
3. McCaffrey, Daniel F., et al. "A tutorial on propensity score estimation for multiple treatments using generalized boosted models." *Statistics in medicine* 32.19 (2013): 3388-3414. [↑](#footnote-ref-4)
4. Austin, Peter C. "An introduction to propensity score methods for reducing the effects of confounding in observational studies." *Multivariate behavioral research* 46.3 (2011): 399-424. [↑](#footnote-ref-5)
5. Hernán MA, Robins JM (2020). Causal Inference: What If. Boca Raton: Chapman & Hall/CRC. [↑](#footnote-ref-6)
